# The effect of smart pillboxes on TB stigma among adults in a cluster-randomised TB treatment trial

**DOI:** 10.5588/ijtldopen.25.0113

**Published:** 2025-10-10

**Authors:** L. Jennings, N. Maraba, R. Mukora, P. Hippner, K. Velen, C. Orrell, S. Charalambous, K. Fielding

**Affiliations:** ^1^ Desmond Tutu HIV Centre, Institute of Infectious Disease and Molecular Medicine and Department of Medicine, University of Cape Town, Cape Town, South Africa;; ^2^ The Aurum Institute, Johannesburg, South Africa;; ^3^ School of Public Health, University of Witwatersrand, Johannesburg, South Africa;; ^4^ London School of Hygiene & Tropical Medicine, London, UK;; ^5^ Health Economics and Epidemiology Research Office, Faculty of Health Sciences, University of the Witwatersrand, Johannesburg, South Africa.

**Keywords:** tuberculosis, South Africa, stigma, electronic pillboxes

## Abstract

**BACKGROUND:**

TB stigma has been shown to result in delayed health-seeking behaviours and treatment initiation. Few studies have quantitatively described stigma during treatment. As part of the TB Mate trial, we summarise TB stigma at treatment start and the effect of the intervention on stigma in follow-up.

**METHODS:**

In the TB Mate trial, we conducted a cluster-randomised trial in 18 primary health care facilities from three provinces in South Africa to evaluate the use of alarmed electronic pillboxes in drug-sensitive TB on treatment adherence. We administered a questionnaire, measuring five TB stigma domains, at baseline, 6 months, and 18 months. We conducted a sub-analysis of these stigma data.

**RESULTS:**

Overall, 2,469/2,657 adults with TB enrolled had a baseline stigma questionnaire. At baseline, reporting experience of stigma or internalised stigma was low (≤3%), whereas disclosure of TB status outside of the household was common (42.3%; 1,045/2,469). Prevalence of experiencing stigma remained low at the end of treatment. Disclosure increased at 6 months in the intervention (40%–64%) and standard of care arms (44.7%–56%), though was similar by arm (adjusted prevalence ratio 2.55 [95% confidence interval: 0.50–12.90]).

**CONCLUSION:**

The overall prevalence of TB stigma, in domains other than disclosure, in our study population was low. There was no evidence that stigma increased with use of an alarmed smart pillbox.

South Africa has one of the highest TB burdens in the world, with an estimated incidence of 468 per 100,000 population in 2022, and HIV prevalence among people with TB of 54%.^1^ Stigma related to TB has been described worldwide and consists of several domains, including internalised, experienced, and anticipated or perceived stigma.^2^ Combating TB stigma and discrimination is one of the World Health Organization’s (WHO) 10 priority recommendations for ending TB.^3^ Despite this, TB stigma in South Africa is not well understood. While qualitative research has extensively described TB stigma in South Africa, it cannot be used to determine the prevalence.^4,5^ Many scales and tools for the measurement of TB stigma exist, but there is a lack of consensus on which tools should be used and which domains of stigma should be covered.^6–8^ To date, only one TB stigma scale, which covers the domain of perceived stigma, has been validated for use in South Africa.^7^ However, qualitative exploration of this tool found that it overestimated the prevalence of stigma.^9^ Other tools, such as the ZAMSTAR and Stop TB Partnership tools, cover multiple domains of stigma.^10,11^ A systematic review has shown that perceived TB stigma results in delayed health-seeking behaviours and treatment initiation^12^; however, this effect has not always been shown^13,14^ and the strength of these associations has been difficult to quantify.^2^

Digital adherence technologies (DATs), such as smart pillboxes and video-supported therapy, have increasingly been evaluated as an approach to improve treatment adherence and outcomes among people with TB. DATs have a conditional recommendation from WHO.^15–17^ There is a concern, however, that these technologies may have an impact on the privacy and confidentiality of people with TB, which may result in increased stigma.^17,18^ A scoping review of contextual factors influencing implementation of DATs among people with TB found that DATs both lowered and increased stigma, with some finding it provided more privacy and others finding it increased visibility of TB treatment.^17^

As a sub-analysis of a large cluster-randomised trial of smart pillboxes conducted in South Africa among people with drug-sensitive TB, we administered the ZAMSTAR TB stigma questionnaire at three timepoints: baseline (treatment start), 6 months (end of treatment), and 18 months. This study aimed to 1) describe the prevalence of TB stigma at treatment start and 2) explore whether the DAT intervention increased TB stigma at 6 and 18 months, versus routine care, among those who were cured or completed treatment.

## METHODS

These data are from a cluster-randomised trial conducted in 18 primary health care facilities (clusters) from three provinces (Gauteng, KwaZulu-Natal, and Western Cape) in South Africa between May 2019 and February 2022.^19^ Clusters were allocated to the intervention or standard of care arm (1:1), using unstratified randomisation. The study enrolled adults and children aged 2–17 years with drug-sensitive TB, who had initiated treatment within the past 14 days at the health care facilities. All participants/caregivers were issued a smart pillbox at study enrolment, which recorded pillbox openings in real-time. In the intervention arm, the device had daily reminder alarms, which sounded at a specified time as reminders to participants to take their medication, and visual appointment reminders in the form of coloured lights indicating when clinic visits were near or due. The devices were monitored in real-time by research staff, and participants received either a text message, a phone call, or a home visit, depending on the number of times the box was not opened. In the standard of care arm, the devices were in silent mode and were not monitored in real-time. Participants were dispensed medication by clinic staff under routine care and seen by research staff at these routine visits for additional data collection for the trial. At study enrolment, socio-demographic data were collected by self-report. The treatment outcome was abstracted from the TB register. A research follow-up visit at the health facility was planned to coincide with the end of treatment (6 months) to collect a sputum specimen for culture to enhance trial endpoint measurement. In addition, participants whose treatment outcome was cured or completed treatment were followed up by research staff until 18 months after study enrolment to measure TB recurrence. The trial’s primary outcome was treatment adherence using a proxy of pillbox openings, in both arms. Secondary outcomes included poor end of treatment outcome (death, lost to follow-up, and treatment failure) and a combined measure of poor end of treatment outcome or TB recurrence by 18 months. The DAT intervention improved treatment adherence, and secondary outcomes were similar by arm.^20^

### Stigma scale

We used the TB stigma scale developed from the ZAMSTAR study in South Africa and Zambia.^10^ This tool was chosen due to its recent development and assessment in sub-Saharan Africa at the time the TB Mate trial was being planned. The stigma scale aimed to address gaps in existing TB scales and to ensure coverage of the following five domains of TB stigma: experience with social exclusion (four questions), experience of being made fun of (three questions), experience with health-setting stigma (one question), internalised stigma (one question), and disclosure of TB status (one question). Each of the five domains was recorded as a binary outcome, with social exclusion and experience of being made fun of, assigned a positive score if at least one question was answered yes (Table 1). Domains were analysed separately. The questionnaire was administered by research staff to all study participants aged 15 years and above at baseline (start of TB treatment), 6 months (end of TB treatment), and 18 months (end of study). For the latter, this was restricted, by design, to those who had cured/completed treatment, in order to measure TB recurrence. The 6-month end of treatment measure coincided with the routine end of treatment clinic visit, and in order not to interfere with the measurement of trial secondary treatment outcomes, no attempt was made by research staff to actively follow up participants who had not attended this clinic visit. Therefore, stigma data were unlikely to have been measured for those that had been transferred out to another clinic, died, or been lost to follow-up on treatment.

**Table 1.** Stigma tool.^**A**^

Since you have fallen ill with TB, have you experienced any of the following	
Social exclusion domain	At least one of
1. Been excluded from a social gathering	Yes/No
2. Abandoned by spouse/partner	Yes/No
3. Isolated by your household	Yes/No
4. Your children or family have been isolated/shunned	Yes/No
Been made fun of domain	At least one of
5. Lost respect or standing in the community	Yes/No
6. Been teased, insulted, or sworn at	Yes/No
7. Been gossiped about	Yes/No
Health-setting stigma domain	
8. Been treated worse than patients with other diseases by health staff	Yes/No
Internalised stigma domain	
9. Unclean or dirty because of your TB	Yes/No
Disclosure domain	
10. Did you tell or have you told anyone outside of your household about your TB diagnosis?	Yes/No

A Bond et al.^10^

### Analysis population

We restricted analyses to trial participants aged 18 years and above with at least the baseline stigma questionnaire completed. Analysis of stigma scores at 6 and 18 months was limited to those who had a treatment outcome of cured or completed treatment at 6 months.

### Statistical analysis

All analyses were conducted in Stata (version 18).^21^ At the 6 and 18 month follow-up visits, the five domains were summarised by study arm, restricted to those that had cured or completed treatment. The effect of the study arm on the 6- and 18-month stigma scores was analysed at the cluster level due to the small number of clusters.^22^ The effect estimate was the prevalence ratio (PR) based on the natural logarithm-transformed prevalences, compared by study arm across clusters using a *t* test. For clusters with zero events, 0.5 was added to the numerator for all clusters. An adjusted analysis was also conducted, controlling for imbalances of individual-level variables at baseline, using a two-stage approach, where there was a reasonable number of events. The first stage used logistic regression at the individual level including all baseline covariates that had imbalance by study arm, excluding arm; observed and expected number of outcomes were generated for each cluster. At the second stage, the log of the cluster-level residual (observed/expected number of events) was compared by study arm across clusters using a *t* test. Analysis of the stigma domains’ experience with social exclusion, health-setting stigma, and internalised stigma was not attempted due to a small number of events. The Stata ‘clan’ command was used for this analysis.^23^

### Ethical statement

This study was approved by the University of Witwatersrand (180705), the University of Cape Town Human Research Ethics Committee (452/2018), and the London School of Hygiene & Tropical Medicine (16107). Permission to conduct the study in health facilities was obtained from local health authorities. Written informed consent was obtained from study participants prior to data collection.

## RESULTS

**Figure 1.**
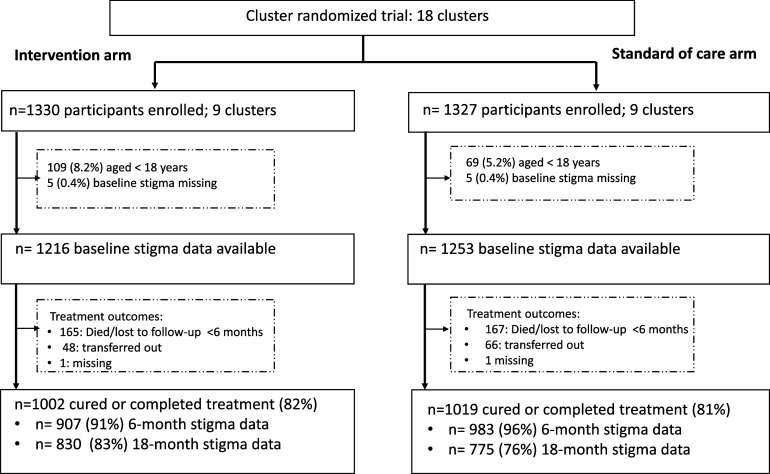
CONSORT flow chart of participants’ disposition throughout study participation.

Overall, 2,657 persons with TB enrolled into the TB Mate trial, of whom 178 (6.7%) were aged <18 years and 10 had missing baseline stigma questionnaire, leaving 2,469 (1,216 and 1,253 in the intervention and standard of care arms, respectively) participants contributing to subsequent analyses (Figure 1). Of these participants, 37% (918/2,469) were female, the median age was 36 years (interquartile range 29–46 years), and 44%, 28%, and 28% were HIV negative (1,059/2,397), positive not on ART (677/2,397), and positive on ART (661/2,397), respectively (Table 2). At the end of treatment, 82% (2,021/2,469) of participants were defined as cured or completed treatment, 13% (332/2,469) were classified as having poor treatment outcome (treatment failure, died, or lost to follow-up), 5% (114/2,469) had been transferred out to another facility, and two participants had missing end of treatment outcome (Figure 1).

**Table 2.** Baseline characteristics by study arm.

		Intervention	SOC	Total
		n = 1,216	n = 1,253	n = 2,469
Country or origin	South Africa	1,184 (97.4%)	1,189 (94.9%)	2,373 (96.1%)
Sex	Male	783 (64.4%)	768 (61.3%)	1,551 (62.8%)
	Female	433 (35.6%)	485 (38.7%)	918 (37.2%)
Ethnicity	Black African	1,089 (89.6%)	1,194 (95.3%)	2,283 (92.5%)
Age, years	Median (Q1, Q3)	37 (29, 47)	36 (29, 46)	36 (29-46)
Education	≤Grade 7	195 (16.0%)	238 (19.0%)	433 (17.5%)
	Grade 8–11	536 (44.1%)	527 (42.1%)	1,063 (43.1%)
	≥Grade 12	485 (39.9%)	488 (38.9%)	973 (39.4%)
Marital status	Single	829 (68.2%)	946 (75.5%)	1,775 (71.9%)
	Married/cohab	324 (26.6%)	246 (19.6%)	570 (23.1%)
	Divorced/widowed	63 (5.2%)	61 (4.9%)	124 (5.0%)
HIV/ART status	Negative	610 (50.2%)	449 (35.8%)	1,059 (42.9%)
	Pos, not on ART	278 (22.9%)	399 (31.8%)	677 (27.4%)
	Pos, on ART	268 (22.0%)	393 (31.4%)	661 (26.8%)
	Unknown	60 (4.9%)	12 (1.0%)	72 (2.9%)
Previous TB	Yes	307 (25.2%)	295 (23.5%)	602 (24.4%)
TB diagnosis	Bacteriological positive	997 (82.1%)	892 (71.3%)	1,889 (76.6%)
Province	Gauteng	431 (35.4%)	343 (27.4%)	774 (31.3%)
	KwaZulu-Natal	356 (29.3%)	434 (34.6%)	790 (32.0%)
	Western Cape	429 (35.3%)	476 (38.0%)	905 (36.7%)
Stigma domains				
Social exclusion	Yes	15 (1.2%)	26 (2.1%)	41 (1.7%)
Made fun of	Yes	25 (2.1%)	26 (2.1%)	51 (2.1%)
Health-setting stigma	Yes	3 (0.2%)	9 (0.7%)	12 (0.5%)
Internalised stigma	Yes	37 (3.0%)	23 (1.8%)	60 (2.4%)
Disclosure	Yes	544 (44.7%)	501 (40.0%)	1,045 (42.3%)

‘Social exclusion’ any of: being excluded from a social gathering; abandoned by spouse/partner; isolated by their household; their children or family have been isolated/shunned; ‘Made fun of’ any of: lost respect or standing in the community; been teased, insulted or sworn at; been gossiped about; ‘Health-setting stigma’: been treated worse than patients with other diseases by health staff; ‘Internalised stigma’: felt unclean or dirty because of your TB; ‘Disclosure’: told anyone outside of their household about their TB diagnosis?

SOC = standard of care; Q1 = lower quartile; Q3 = upper quartile; cohab = cohabiting; Pos = positive.

At baseline, the percentage of participants reporting experience of social exclusion, being made fun of, health-setting stigma, and internalised stigma was low (≤3%), and similar by arm. Overall 42.3% (1,045/2,469) of participants reported disclosing their TB diagnosis to anyone outside of their household: 40.0% in the standard of care arm and 44.7% in the intervention arm. Experience of social exclusion and experience of being made fun of were more commonly reported among participants enrolled in KwaZulu-Natal (3% [24/790] and 4% [32/790], respectively) than Gauteng (<1% [6/774] and <1% [4/774], respectively) or Western Cape (1% [11/905] and 2% [15/905], respectively). Disclosure was most common in Western Cape (61%, 553/905), followed by KwaZulu-Natal (36%, 284/790) and Gauteng (27%, 208/774) – see Supplementary Data Table S1.

At the end of treatment, 81.9% (2,021/2,469) had a treatment outcome of cured or completed treatment, and of these 93.5% (1,890/2,021) and 79.4% (1,605/2,021) had completed the stigma questionnaire, at 6 and 18 months, respectively. Non-attendance at the 18-month visit (resulting in missing stigma data at this visit) was less common for those with education grade 12 and above, HIV negative or HIV positive on ART, the intervention arm, and participants from Gauteng and KwaZulu-Natal provinces (Supplementary Data Table S3).

**Figure 2.**
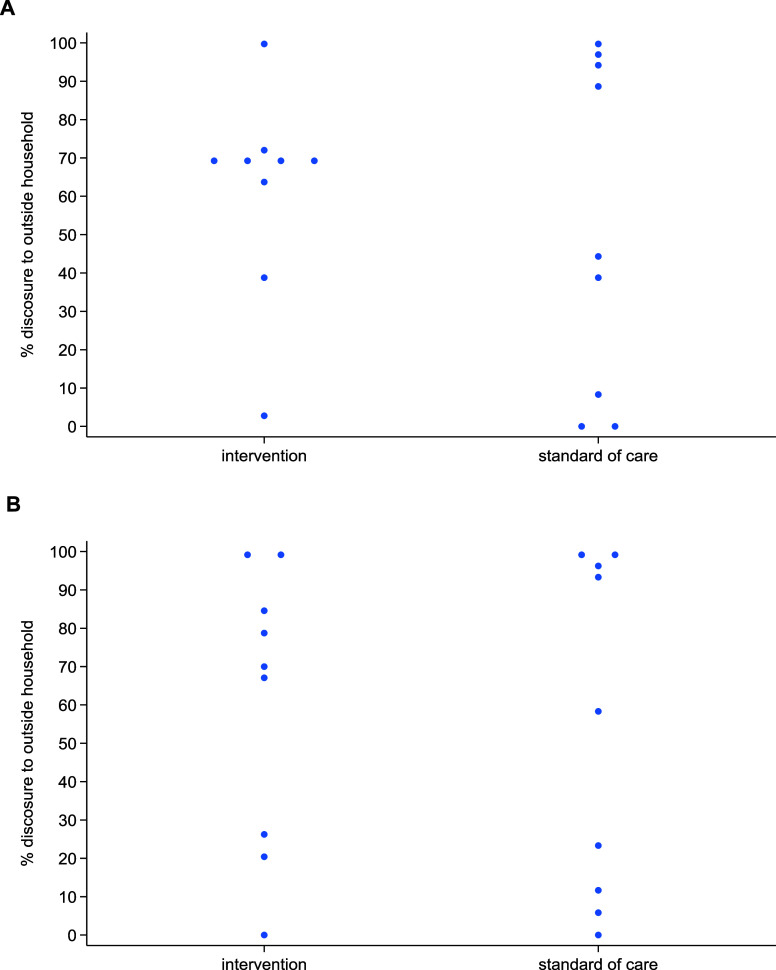
**A:** disclosure at 6 months, at the cluster level (n = 1,890); **B:** disclosure at 18 months, at the cluster level (n = 1,605).

At 6 months, reporting experience of social exclusion, being made fun of, health-setting stigma, and internalised stigma was uncommon (Table 3). Disclosure had increased from baseline: 64% (576/907; geometric mean [GM] of cluster-level proportions 49%) reported disclosure in the intervention arm compared with 56% (547/983; GM of cluster-level proportions 20%) in the standard of care arm, giving an adjusted PR of 2.55 (95% confidence interval [CI] 0.50–12.90), adjusting for age, sex, type of TB diagnosis, ethnicity, marital status, and HIV/anti-retroviral status. There was considerable variability of reporting disclosure by cluster (Figure 2). Participants' experience of being made fun of was higher in those enrolled in KwaZulu-Natal than those in other provinces at 6 months. (6%; 42/605). Disclosure was most common in KwaZulu-Natal (73%, 367/710) (Supplementary Data Table S1B). At 18 months, disclosure was similar by arm with an adjusted PR of 1.36 (95% CI 0.32–5.89).

**Table 3.** Effect of intervention arm on the five stigma domains at end of treatment (6 months) and 18 months, restricted to those who had cured/completed treatment.

	SOC			Intervention			Crude PR (95% CI)
	n (%)	GM, %^**A**^	# clusters with zero outcomes	n (%)	GM, %^**A**^	# clusters with zero outcomes	
At 6 months	N = 983		N = 9	N = 907		N = 9	
Social exclusion	8 (0.8%)	—	6	13 (1.5%)	—	4	—
Made fun of	29 (3.0%)	1.4%	5	44 (4.9%)	3.1%	1	2.29 (0.65–8.06)
Health-setting stigma	2 (0.2%)	—	8	1 (0.1%)	—	8	—
Internalised stigma	11 (1.2%)	—	7	45 (5.0%)	—	6	—
Disclosure	547 (56%)	20%	2	576 (64%)	49%	0	2.46 (0.46–13.2)
At 18 months	N = 775			N = 830			
Social exclusion	3 (0.4%)	—	7	9 (1.1%)	—	4	—
Made fun of	6 (0.8%)	1.1%	5	25 (3.0%)	1.7%	4	1.66 (0.56–4.94)
Health-setting stigma	2 (0.3%)	—	8	7 (0.8%)	—	7	—
Internalised stigma	11 (1.4%)	—	5	38 (4.6%)	—	4	—
Disclosure	434 (56%)	28%	0	519 (63%)	37%	1	1.30 (0.27–6.29)

Analysis restricted to those cured/completed treatment – n = 1,890/2,021 (93.5%) have stigma measured at 6 months; and n = 1,605/2,021 (79.4%) have stigma measured at 18 months. Disclosure at 6 months, adjusted PR 2.55 (95% CI: 0.50–12.90); disclosure at 18 months, adjusted PR 1.36 (95% CI: 0.32–5.89). Adjustment was conducted for age, sex, type of TB diagnosis, ethnicity, marital status, and HIV/anti-retroviral status.

PR = prevalence ratio; CI = confidence interval; SOC = standard of care; GM = geometric mean; # = number.

A 0.5 added to numerator for all cluster-level summaries due to zero cells.

## DISCUSSION

This sub-analysis of a large cluster-randomised TB adherence study using smart pillboxes aimed to describe (among adults with DS-TB) the prevalence of TB stigma at treatment start and the effect of the alarmed DAT intervention on stigma at 6 and 18 months from treatment start. We found that the prevalence of the TB stigma domains of experience of social exclusion, being made fun of, health-setting stigma, and internalised stigma was low at baseline, in both arms. There was no effect of the alarmed DAT on reported stigma in these domains at the end of treatment, among the subgroup who had cured/completed treatment. Stigma domains were highly variable across clusters.

Bresenham et al.^8^ described an association between health district and stigma and, in our study, we found that disclosure rates at 6 months showed great variability by cluster. Although this finding may be due to data collection discrepancies across sites, the influence of local context on stigma has been emphasised in multiple studies^10,24–26^ and highlights the need for interventions that are multi-faceted and adaptable to different settings. Bond et al.^10^ found low prevalence of experience of social exclusion, being made fun of, and internalised stigma in South Africa, which is consistent with our findings. Low prevalence of health-setting stigma has also been described elsewhere.^10,27^ Similarly to our study, Bond et al.^10^ also found that disclosure of TB status was high at baseline and, as they noted, the relationship between stigma and disclosure is complex. While disclosure can indicate that a participant felt comfortable to do so, it can also increase the risk of the participant experiencing stigma. We did not see a difference in stigma in the intervention arm, in which the pillboxes used had audio and visual alarms, and the control group, in which the pillboxes were in silent mode. These results are not surprising given a review of contextual factors influencing implementation of DATs in TB, which showed that DATs are likely to both increase and decrease stigma depending on the context in which they are being used.^17^ This is supported by the great variability in stigma seen by cluster.

There are several limitations to this study. Baseline stigma assessment was conducted primarily at the time of starting TB treatment with individuals not having yet had the experience of taking treatment at home. It may have been more appropriate to assess baseline stigma within the first few weeks of treatment. Our analyses at 6 and 18 months were limited to participants who were cured or who had completed treatment, limiting the generalisability of our findings; we cannot comment on stigma in those who had treatment failure, died, or were lost to follow-up. This group is important to understanding the effects of stigma, but our study design did not allow us to measure stigma at these time points. Although the baseline characteristics were similar in both arms, variability of TB disclosure in clusters indicates that there are factors influencing this domain which require further study. High variability between clusters and low prevalence of stigma outcomes resulted in low power to detect differences between arms. Additionally, this was a sub-analysis of a study powered for adherence outcomes^20^ and was therefore not powered to detect differences in stigma outcomes by study arm.

Our findings show that the prevalence of TB stigma, in domains other than disclosure, in our study population was low; however, the lack of standardised tools makes this difficult to compare across studies. There was no evidence that using the DAT in active versus silent mode increased stigma in the domains of experience of social exclusion, being made fun of, health-setting stigma, or internalised stigma in people who were cured or completed TB treatment. Future research should focus on the standardisation of TB stigma measures to properly assess the impact of stigma-related interventions. Measures need to be adaptable as the prevalence of TB stigma varies by context. In addition, future studies should assess the association between TB stigma and treatment outcomes.

## Supplementary Material


