# Supplementary material for: The effect of smart pillboxes on TB stigma among adults in a cluster-randomised TB treatment trial
Source: IJTLD Open. 2025 Oct 10;2(10):583–9. doi: 10.5588/ijtldopen.25.0113 (PMC12517265; doi:10.5588/ijtldopen.25.0113)
Supplement: Supplementary file 1 [file ijtldopen25-0113_supplementarydata1.pdf]

SUPPLEMENT

Table S1a: components of the stigma score at baseline, by province of enrolment (n=2469)

**Since you have fallen ill with TB, have you experienced any of the following:**	**Gauteng n (%)**		**KwaZulu-Natal n (%)**		**Western Cape n (%)**		**Overall n (%)**	
n	774		790		905		2,469	
**Experience of social exclusion**								
Been excluded at social gathering	4	0.5%	15	1.9%	10	1.1%	29	1.2%
Abandoned by spouse/partner	2	0.3%	5	0.6%	0	0.0%	7	0.3%
Isolated by your household	0	0.0%	13	1.6%	1	0.1%	14	0.6%
children or family have been isolated/shunned	0	0.0%	5	0.6%	0	0.0%	5	0.2%
**At least one**	**6**	**0.8%**	**24**	**3.0%**	**11**	**1.2%**	**41**	**1.7%**
**Experience of being made fun of**								
Lost respect or standing in the community	1	0.1%	17	2.2%	7	0.8%	25	1.0%
Been teased, insulted or sworn at	1	0.1%	25	3.2%	4	0.4%	30	1.2%
Been gossiped about	3	0.4%	31	3.9%	12	1.3%	46	1.9%
**At least one**	**4**	**0.5%**	**32**	**4.1%**	**15**	**1.7%**	**51**	**2.1%**
**Experience of health setting stigma**								
Been treated worse than patients with other diseases by health staff	**0**	**0.0%**	**10**	**1.3%**	**2**	**0.2%**	**12**	**0.5%**
**Internalised stigma (Since you were diagnosed with TB have you felt)**								
Unclean or dirty because of your TB?	**29**	**3.7%**	**22**	**2.8%**	**9**	**1.0%**	**60**	**2.4%**
**Disclosure**								
Have you told anyone outside of your household about your TB diagnosis?	**208**	**26.9%**	**284**	**35.9%**	**553**	**61.1%**	**1,045**	**42.3%**

Table S1b: components of the stigma score at 6 months, by province of enrolment, and baseline data restricted to same sample who had treatment outcome of cured/completed treatment (n=1890)

	Gauteng		KwaZulu-Natal		Western Cape		Overall	
	n	%	n	%	n	%	n	%
Total	609		605		676		1890	
**6 month data**								
Experience of social exclusion	11	1.8%	10	1.6%	0	0%	21	1.1%
Experience of being made fun of	12	2.0%	42	6.9%	19	2.8%	73	3.9%
Experience of health setting stigma	0	0.0%	3	0.5%	0	0.0%	3	0.2%
Internalised stigma	43	7.1%	9	1.5%	5	0.7%	57	3.0%
Disclosure	324	53.2%	441	72.9%	358	52.0%	1,123	59.4%
**Baseline data, restricted to n=1890**								
Experience of social exclusion	4	0.7%	15	2.5%	10	1.5%	29	1.5%
Experience of being made fun of	3	0.5%	19	3.1%	13	1.9%	35	1.8%
Experience of health setting stigma	0		9	1.5%	1	0.1%	10	0.5%
Internalised stigma	18	3.0%	11	1.8%	6	0.9%	35	1.8%
Disclosure	165	27.1%	214	35.4%	413	61.1%	792	41.9%

Table S1c: components of the stigma score at 18 months, by province of enrolment, and baseline data restricted to same sample who had treatment outcome of cured/completed treatment (n=1605)

	**Gauteng**		**KwaZulu-Natal**		**Western Cape**		**Overall**	
	n	%	n	%	n	%	n	%
Total	515		517		573		1605	
**18 month data**								
Experience of social exclusion	4	0.8%	5	1.0%	3	0.5%	12	0.8%
Experience of being made fun of	3	0.6%	18	3.5%	10	1.8%	31	1.9%
Experience of health setting stigma	3	0.6%	6	1.2%	0	0.0%	9	0.6%
Internalised stigma	16	3.1%	31	6.0%	2	0.4%	49	3.0%
Disclosure	222	43.1%	391	75.6%	340	59.3%	953	59.4%
**Baseline data, restricted to n=1605**								
Experience of social exclusion	3	0.6%	12	2.3%	9	1.6%	24	1.5%
Experience of being made fun of	3	0.6%	15	2.9%	8	1.4%	26	1.6%
Experience of health setting stigma	0		6	1.2%	1	0.2%	7	0.4%
Internalised stigma	14	2.7%	9	1.7%	6	1.0%	29	1.8%
Disclosure	145	28.2%	178	34.4%	352	61.4%	675	42.1%

Table S2: cluster-level on the five stigma domains at 6 (n=1890) and 18-months (n=1605) from treatment start

arm	Cluster code	6 months after treatment start socexcl, n	madefun, n	treatbad, n	unclean, n	toldouthh, n	N	18 months after treatment start socexcl, n	madefun, n	treatbad, n	unclean, n	toldouthh, n	N
Intervention	1	0	1	0	0	81	115	0	0	0	0	20	104
	2	1	3	0	0	62	90	0	1	0	1	23	84
	3	1	7	0	2	50	73	1	7	0	3	49	70
	4	3	21	1	0	74	101	3	8	6	19	88	88
	5	0	1	0	5	75	118	2	8	0	2	99	118
	6	0	0	0	0	3	99	0	0	0	0	0	77
	7	0	1	0	0	31	81	1	1	0	0	65	98
	8	6	4	0	0	70	100	2	0	0	0	63	79
	9	2	6	0	38	130	130	0	0	1	13	112	112
SOC	10	2	1	0	5	32	81	2	2	2	2	17	73
	11	0	0	0	0	93	99	0	0	0	0	84	89
	12	0	6	0	0	53	118	1	2	0	5	56	94
	13	5	5	2	7	109	124	0	0	0	3	91	92
	14	0	0	0	0	128	128	0	0	0	0	81	82
	15	0	17	0	0	121	124	0	1	0	0	94	99
	16	0	0	0	0	0	126	0	0	0	0	1	99
	17	0	0	0	0	11	124	0	0	0	0	4	90
	18	1	0	0	0	0	59	0	1	0	1	6	57

socexcl: Social exclusion any of - being excluded from a social gathering; abandoned by spouse/partner; isolated by their household; their children or family have been isolated/shunned
madefun: Been made fun of, any of - lost respect or standing in the community; been teased, insulted or sworn at; been gossiped about
treatbad: Health setting stigma: been treated worse than patients with other diseases by health staff
unclean - Internalised stigma: felt unclean or dirty because of your TB
toldouthh: Disclosure : told anyone outside of their household about their TB diagnosis

Table S3: summary of missing stigma data at 18-months from treatment start, due to lost to follow-up (n=416/2021; 20.6%)

		Missing stigma at 18 months	%	Total	P-value*
Study arm	Intervention	172	17.2%	1,002	0.14
	Standard of care	244	23.9%	1,019	
Country of birth					0.82
	South Africa	401	20.6%	1,943	
	Zimbabwe	6	17.6%	34	
	Mozambique	6	24.0%	25	
	other	3	15.8%	19	
Sex	male	259	20.8%	1,248	0.83
	female	157	20.3%	773	
Age group, years					0.60
	18-19	15	26.8%	56	
	20-29	106	22.0%	482	
	30-39	139	21.1%	660	
	40-49	94	20.4%	460	
	≥50	62	17.1%	363	
Education	Grade ≤7	83	23.6%	352	<0.001
	Grade 8-11	209	24.6%	851	
	Grade ≥12	124	15.2%	818	
Marital status	single	306	21.2%	1,444	
	married/cohabiting	92	19.4%	474	
	Divorced/ widowed	18	17.5%	103	
HIV/ART status	negative	165	18.6%	885	0.022
	Positive -not on ART	142	26.2%	542	
	Positive - on ART	103	19.0%	543	
	unknown	6	11.8%	51	
Previous TB	No	302	19.6%	1,539	0.084
	Yes	114	23.7%	482	
Province	Gauteng	99	16.1%	614	0.036
	KwaZulu- Natal	101	16.3%	618	
	Western Cape	216	27.4%	789	

* based on using robust standard errors to control for clinic-level clustering
